# Age-specific benefits of Vitamin D and its association with mortality

**DOI:** 10.1371/journal.pone.0330959

**Published:** 2025-08-29

**Authors:** Yan Wang, Xueqin Zhang, Xu Wang, Ju Li, Kai Wang

**Affiliations:** 1 Department of Rehabilitation, The affiliated Huaian No.1 People’s Hospital of Nanjing Medical University, Huaian, China; 2 Department of Rheumatology and Immunology, The affiliated Huaian No.1 People’s Hospital of Nanjing Medical University, Huaian, China; Guangdong Nephrotic Drug Engineering Technology Research Center, Institute of Consun Co. for Chinese Medicine in Kidney Diseases, CHINA

## Abstract

**Background:**

Vitamin D is a fat-soluble secosteroid that plays essential roles in calcium homeostasis, bone metabolism, and numerous other physiological processes. Vitamin D deficiency has been associated with increased risk of various diseases and mortality. However, population-based studies examining the relationship between vitamin D and mortality across different age groups remain limited.

**Methods:**

To investigate the correlation between 25-hydroxyvitamin D [25(OH)D] levels, vitamin D status, and mortality in a cohort of 47,478 individuals aged 18–85 years.

**Results:**

Higher 25(OH)D levels were associated with lower mortality risk. Compared to the vitamin D deficiency group, the Hazard ratios (HR) for all-cause mortality were 0.71 (95%CI: 0.66–0.76) in the insufficiency group and 0.64 (95%CI: 0.58–0.70) in the sufficiency group. The association varied by age: strongest in adults aged 40–59 years (HR: 0.74, 95% CI: 0.65–0.85), significant in those ≥60 years (HR: 0.86, 95% CI: 0.82–0.90), but non-significant in those aged 18–39 years. The RCS analysis revealed a non-linear relationship between 25(OH)D and mortality, with significant risk reduction observed between 59.25–261.45 nmol/L for the overall population. The optimal 25(OH)D levels (lowest HR) varied by subgroups: 96.81 nmol/L for the overall population, 102.9 nmol/L for females, 67 nmol/L for ages 40–59, and 104.23 nmol/L for ages ≥60 years, while no significant association was found in ages 18–39 years.

**Conclusion:**

Our findings suggest that Vitamin D are associated with mortality among the whole population. Individuals aged 40–59 may derive potential benefits from vitamin D supplementation.

## Introduction

Vitamin D is crucial for maintaining bone health through its regulation of calcium and phosphate metabolism [1]. It is synthesized in the skin upon UVB radiation exposure and can also be obtained through dietary sources and supplements [2]. Vitamin D deficiency represents a significant global public health concern, affecting a substantial proportion of the population across all age groups [3]. Beyond its classical role in bone metabolism, vitamin D has been recognized for its involvement in multiple physiological processes, including immune function, cardiovascular health, and cell differentiation Several studies have suggested that low levels of 25-hydroxyvitamin D [25(OH)D] might be linked to a heightened susceptibility to diverse chronic ailments, encompassing cardiovascular disease (CVD), autoimmune diseases, diabetes, and cancer [4–8].

Previous research has primarily emphasized studying the connection between deficient vitamin D and specific diseases. However, there is limited evidence on the relationship between mortality and deficient vitamin D in the whole population. Furthermore, it is worth noting that while vitamin D is crucial for maintaining optimal health, excessive intake can also be harmful. Vitamin D intoxication can be observed when 25(OH)D level is greater than 375 nmol/L [2]. Vitamin D intoxication can result in hypercalcemia, kidney stones, and potential organ harm. [9,10] However, literature retrieval found that vitamin D intoxication is rare, which may be related to the high level of 25(OH)D required for vitamin D intoxication. Therefore, it is crucial to identify the optimal levels of 25(OH)D that promote health while avoiding potential harm.

To address these knowledge gaps, we analyzed data from the National Health and Nutrition Examination Survey (NHANES) to investigate the associations between 25(OH)D levels, vitamin D status, and both all-cause and cause-specific mortality in a large population-based study The findings from this analysis will enhance the existing understanding of vitamin D and provide valuable insights for public health initiatives focused on optimizing 25(OH)D levels to enhance health outcomes.

## Materials and methods

### Study population

The NHANES data used in this analysis are publicly available and de-identified, ensuring that ethical approval was not required for this study. This is a retrospective cohort study analyzing data from nine consecutive cycles of the NHANES (2001−2018). NHANES itself is a cross-sectional survey design, but through linkage with National Death Index (NDI) mortality follow-up data, we created a cohort analysis where vitamin D measured during NHANES participation served as baseline measurements. The inception cohort was established by including participants who met the following criteria: (1) age between 18 and 85 years at baseline; (2) had available serum 25(OH)D measurements; and (3) had complete mortality follow-up data. Mortality status was ascertained through linkage to the National Death Index (NDI) records. The NDI is a centralized database of death record information maintained by the National Center for Health Statistics (NCHS). Causes of death were classified according to the International Classification of Diseases, 10th Revision (ICD-10). We analyzed ten major causes of death including diseases of heart, malignant neoplasms, chronic lower respiratory diseases, accidents, cerebrovascular diseases, Alzheimer’s disease, diabetes mellitus, influenza and pneumonia, nephritis/nephrotic syndrome/nephrosis, and all other causes. The NCHS linked NHANES participants to NDI death certificate records using probabilistic matching based on 12 identifiers including social security number, name, birth date, sex, race, state of birth, and state of residence. After applying the inclusion criteria, 47,478 participants formed the final study cohort. Participants entered the cohort at their baseline examination date when their vitamin D were measured. The follow-up period began at each participant’s baseline examination date and continued until either death or the end of follow-up (December 31, 2019), whichever came first. The median follow-up duration was 104 months (maximum 229 months). Participants with missing data on key covariates were handled through multiple imputation (details of the imputation process are provided in S2 Fig). After performing Cox regression with stepwise selection, all remaining variables were retained in the final analysis (S1 Table).

### Statistical analyses

The investigation of mortality in relation to 25(OH)D concentrations was conducted through a Cox proportional hazards regression framework. vitamin D status (the definition see Table 1) was incorporated into the model as a categorical variable [3]. Additionally, to elucidate the complex interplay between vitamin D parameters and mortality outcomes, stratified analyses were implemented, examining the relationships among 25(OH)D concentrations, vitamin D status, and all-cause mortality. These analyses incorporated key demographic variables, with particular emphasis on age-specific and sex-specific patterns. Furthermore, vitamin D was incorporated into the analytical model as a standardized continuous variable, expressed in terms of standard deviation (SD) units. The quantification of mortality risk differentials across subgroups was achieved through the computation of hazard ratios (HR) with their corresponding 95% confidence intervals (CI), thereby enabling systematic comparison of mortality patterns among distinct population segments.

**Table 1:** Demographic characteristics of individuals according to vitamin D status, weighted.

Characteristic	Deficiency	Insufficiency	Sufficiency	p-value
	**25(OH)D < 50 nmol/L**	**50 nmol/L ≤ 25(OH)D ≤ 75 nmol/L**	**25(OH)D > 75 nmol/L**	
	**(N = 56154827)**	**(N = 82467947)**	**(N = 73551541)**	
Sex = female (%)	29901985 (53.2)	38472326 (46.7)	41331470 (56.2)	**< 0.001**
Age (years)	420.00 (290.00, 550.00)	440.00 (310.00, 570.00)	50.00 (350.00, 640.00)	**< 0.001**
Race (%)				**< 0.001**
Mexican American	7966346 (14.2)	7792803 (9.4)	2315024 (3.1)	
Other Hispanic	3969396 (7.1)	5338760 (6.5)	2207546 (3.0)	
Non-Hispanic White	23661783 (42.1)	58004491 (70.3)	62826962 (85.4)	
Non-Hispanic Black	14904075 (26.5)	5613794 (6.8)	2527090 (3.4)	
Other Race	5653227 (10.1)	5718099 (6.9)	3674919 (5.0)	
25(OH)D (nmol/L)	38.55 (30.75, 44.70)	62.52 (56.47, 68.35)	90.10 (81.15, 104.45)	**< 0.001**
Months of follow-up	110.00 (60.00, 1670.00)	110.00 (590.00, 1680.00)	940.00 (480.00, 1460.00)	**< 0.001**
Annual household income (%)				**< 0.001**
Under $44,999	29788325 (53.0)	33958401 (41.2)	25915489 (35.2)	
$45,000 to $74,999	17113263 (30.5)	28146205 (34.1)	22802742 (31.0)	
$75,000 and Over	9253239 (16.5)	20363341 (24.7)	24833310 (33.8)	
Marital status (%)				**< 0.001**
Married/cohabiting	30761758 (54.8)	53533193 (64.9)	48961605 (66.6)	
Widowed/divorced/separated	10784853 (19.2)	13624685 (16.5)	13886892 (18.9)	
Never married	14608216 (26.0)	15310069 (18.6)	10703044 (14.6)	
Education level (%)				**< 0.001**
Under high school	12877933 (22.9)	14454411 (17.5)	9120492 (12.4)	
High school or equivalent	14130861 (25.2)	19882416 (24.1)	17459349 (23.7)	
Above high school	29146033 (51.9)	48131120 (58.4)	46971700.1 (63.9)	
BMI	29.20 (24.90, 34.43)	27.80 (24.28, 32.20)	26.50 (23.30, 30.48)	**< 0.001**
Diabetes (%)				**< 0.001**
No	49286541 (87.8)	74435373 (90.3)	65591365 (89.2)	
Borderline	1065798 (1.9)	1336311 (1.6)	1534347 (2.1)	
Yes	5802488 (10.3)	6696263 (8.1)	6425829 (8.7)	
Hypertension = Yes (%)	17047895 (30.4)	23274780 (28.2)	23886157 (32.5)	**< 0.001**
Weak/failing kidneys = Yes (%)	1385506 (2.5)	1502178.3 (1.8)	2206558 (3.0)	**< 0.001**
Total Cholesterol (mmol/L)	4.86 (4.22, 5.64)	4.97 (4.27, 5.66)	4.99 (4.32, 5.72)	**< 0.001**

Note: for 25(OH)D, 1 ng/mL ≈ 2.5 nmol/L.

Abbreviations: 25(OH)D = 25-hydroxyvitamin D; BMI = Body mass index.

In order to delineate potential nonlinear associations between mortality outcomes and 25(OH)D levels, restricted cubic splines (RCS) were integrated into the Cox regression framework, implementing a 5-knot configuration. The analytical framework incorporated comprehensive covariate adjustment to mitigate confounding influences, encompassing demographic parameters (sex, age, race), socioeconomic indicators (annual household income, marital status, educational attainment), anthropometric measurements (body mass index, BMI), lifestyle factors (physical activity patterns, smoking behavior, alcohol consumption), and clinical variables (diabetes status, hypertension diagnosis, renal function impairment, total cholesterol levels).

The statistical analyses were executed using R statistical software (version 4.1.0), employing specialized analytical packages. Specifically, the unweighted Cox proportional hazards regression model was implemented through the “cph()” function within the “rms” package, while the unweighted RCS analysis utilized the “coxph()” function from the “survival” package. For weighted analyses, the statistical framework incorporated the “svydesign()” and “svycoxph()” functions from the “survey” package to construct both the weighted Cox proportional hazards regression model and RCS analyses.

## Results

### Study individuals

A total of 47,478 individuals were included in the study (S1 Fig). We first conducted a missing data check and imputation on the dataset. Covariates with a missing value proportion greater than 5% were deleted (S2 Fig). After performing Cox regression with stepwise to include the remaining variables for analysis, all remaining variables were retained (S1 Table).

The weighted baseline demographic and clinical characteristics, stratified by vitamin D status, are comprehensively documented in Table 1 and S2 Table (corresponding unweighted analyses are presented in S3 and S4 Tables). Analysis of demographic patterns revealed distinct vitamin D status distributions across ethnic groups, with Non-Hispanic White participants demonstrating more favorable vitamin D profiles relative to other ethnic categories. Similarly, individuals in married or cohabiting relationships exhibited superior vitamin D status compared to alternative relationship classifications.

Further examination of socioeconomic and clinical parameters unveiled significant correlational patterns with 25(OH)D concentrations. Specifically, positive correlations were observed between 25(OH)D levels and multiple variables, including educational attainment, annual household income, and total cholesterol measurements. Conversely, BMI demonstrated an inverse relationship with 25(OH)D concentrations (S3 Fig).

### Kaplan-Meier survival probability and cumulative hazard

**Fig 1:**
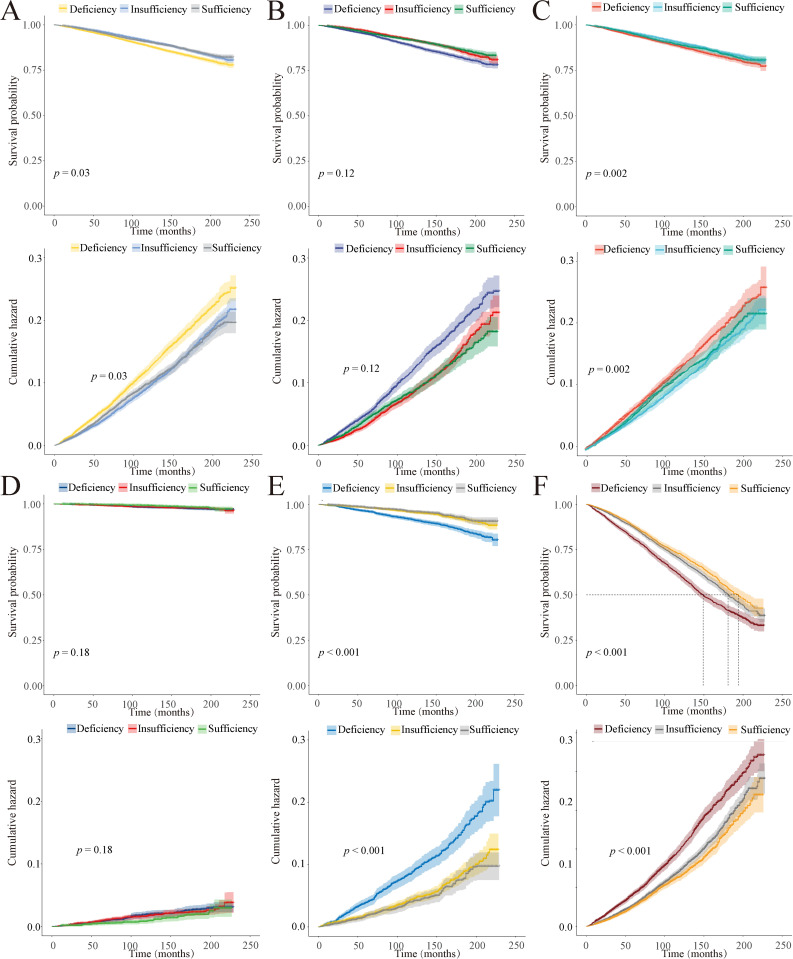
Kaplan-Meier survival probability for all-cause mortality in all individuals (A), female individuals (B), male individuals (C), individuals aged 18-39 (D), individuals aged 40-59 (E), and individuals aged 60 or older (F).

During a follow-up period of up to 229 months (median of 104 months), we identified 6,231 deaths (weighted to 20,644,280). Fig 1 and S4 Fig illustrates the survival probability and cumulative hazard of 25(OH)D levels in relation to all-cause mortality in different populations. As time progresses, the vitamin D deficiency group exhibits the lowest survival probability and highest cumulative hazard. In females and the 18–39 age group, no significant correlation is observed between 25(OH)D levels and survival probability or cumulative hazard. However, among males and the 39 and above age group, the vitamin D deficiency group demonstrates the lowest survival probability and highest cumulative hazard.

**Fig 2:**
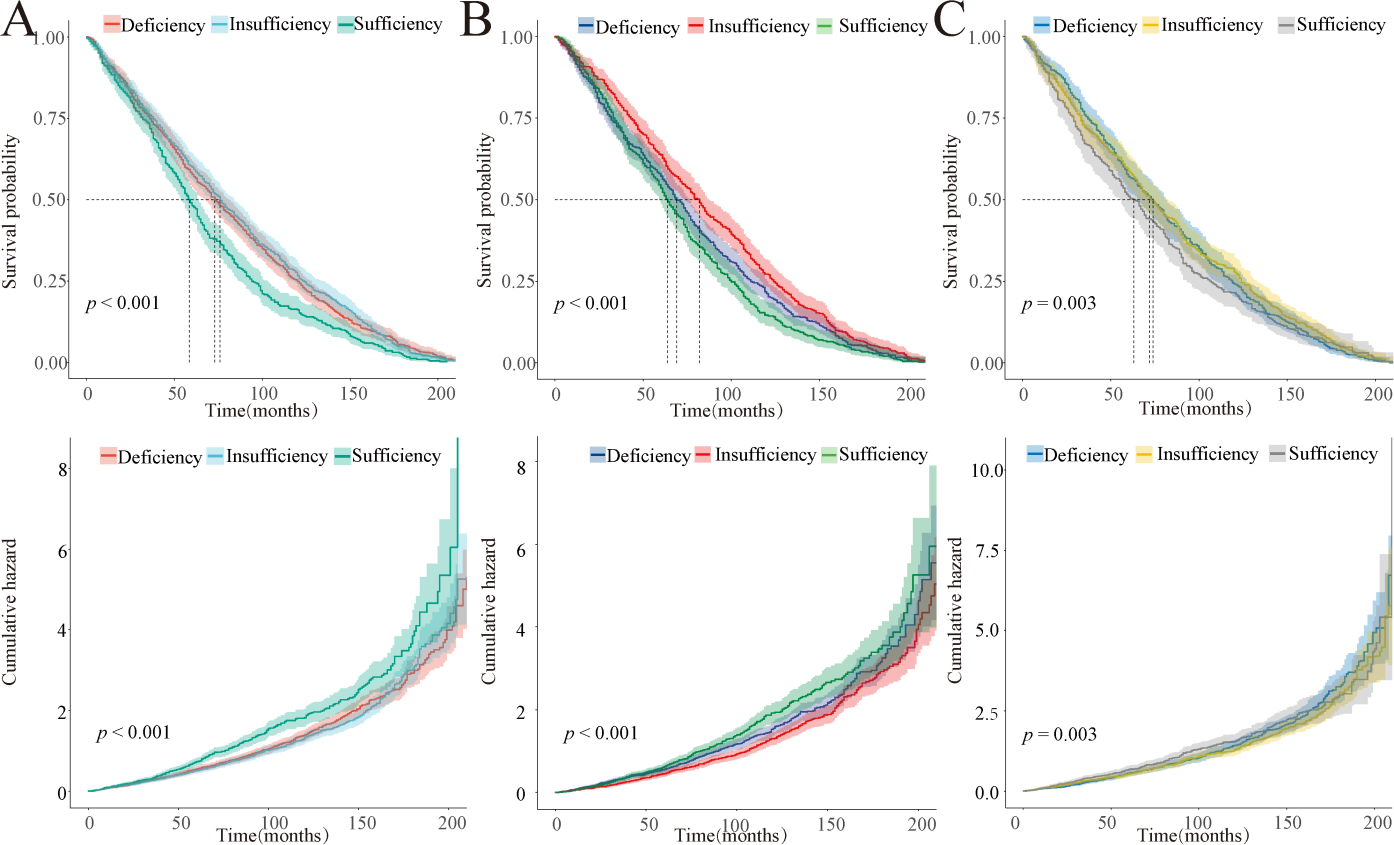
Kaplan-Meier survival probability and cumulative hazard for cause-diseases of heart mortality (A), cause-malignant neoplasms mortality (B), and cause-all other causes mortality (C) in all individuals.

Furthermore, we conducted a more detailed analysis of the survival probability and cumulative hazard of 25(OH)D levels in relation to cause-specific mortality across the whole population. The vitamin D deficiency group exhibits the lowest survival probability and highest cumulative hazard in cause-specific mortalities from diseases of the heart, malignant neoplasms, and all other causes (Fig 2). However, in the remaining cause-specific mortalities, no significant correlation is observed between 25(OH)D levels and survival probability or cumulative hazard (S5 and S6 Figs).

### Cox proportional hazards regression

**Fig 3:**
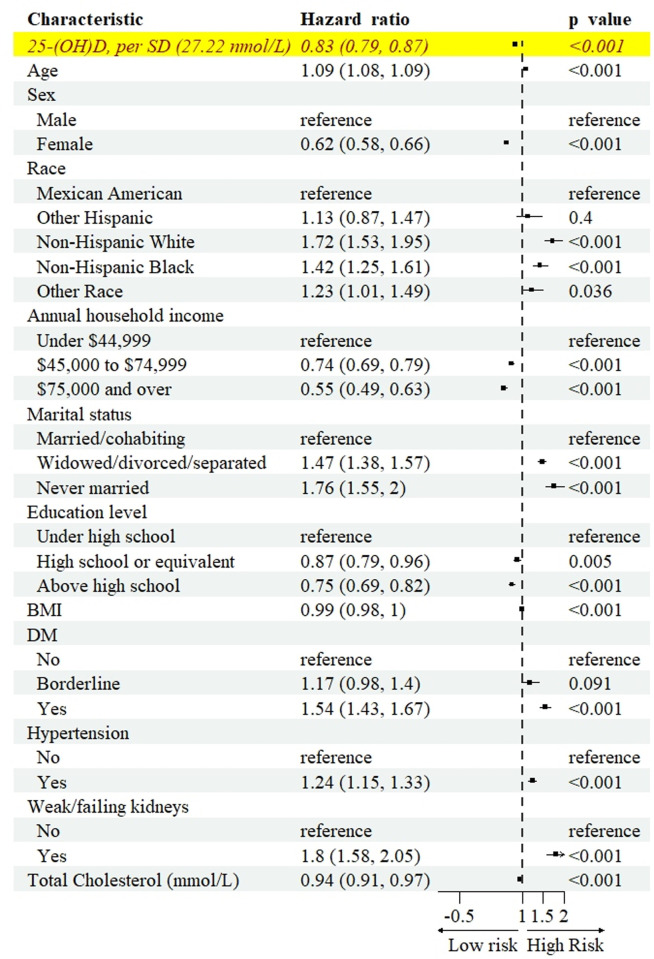
Hazard ratios (95% CI) of 25(OH)D and all covariates, weighted. Forest plot showing hazard ratios and 95% CIs for 25(OH)D and all demographic, socioeconomic, and clinical covariates included in the multivariable Cox proportional hazards regression model. 25(OH)D was analyzed as a standardized continuous variable. All models were adjusted for age, sex, race, annual household income, marital status, education level, BMI, diabetes, hypertension, weak/failing kidneys, and total cholesterol. Abbreviations: 25(OH)D = 25-hydroxyvitamin D; BMI = Body mass index, CI = Confidence interval.

Fig 3 demonstrates the relationship between 25(OH)D levels, all covariates, and all-cause mortality. We observed a strong inverse correlation between all-cause mortality and 25(OH)D levels, with a HR of 0.83 (weighted, 0.79 to 0.87) for per SD (27.22 nmol/L) prediction (unweighted results see S7 Fig). Table 2 demonstrates the relationship between cause-specific mortality and 25(OH)D levels. Regarding specific causes of mortality, the HR per SD for cause-diseases of heart mortality was 0.79 (weighted, 0.73 to 0.86). For cause-malignant neoplasms mortality, the HR per SD was 0.86 (weighted, 0.78 to 0.94). Cause-chronic lower respiratory diseases mortality had a HR of 0.81 (weighted, 0.68 to 0.97) for per SD. Cause-influenza and pneumonia mortality had a HR of 0.71 (weighted, 0.55 to 0.93) for per SD. The HR per SD for cause-all other causes mortality was 0.79 (weighted, 0.73 to 0.86). In contrast, there was no correlation between 25(OH)D levels and cause-accidents mortality, cause-cerebrovascular diseases mortality, cause-Alzheimer’s disease mortality, cause-diabetes mortality, or cause-nephritis, nephrotic syndrome and nephrosis mortality.

**Table 2:** Hazard ratios (95% CI) of all-cause mortality according to 25(OH)D levels.

	Unweighted, SD = 26.74		Weighted, SD = 27.22	
Underlying Leading Cause of Death	HR per SD (95% CI)	p-value	HR per SD (95% CI)	p-value
Cause specific mortality				
Diseases of heart	0.81 (0.77, 0.86)	**< 0.001**	0.79 (0.73, 0.86)	**< 0.001**
Malignant neoplasms	0.88 (0.82, 0.93)	**< 0.001**	0.86 (0.78, 0.94)	**0.001**
Chronic lower respiratory diseases	0.86 (0.76, 0.98)	**0.021**	0.81 (0.68, 0.97)	**0.021**
Accidents	1.01 (0.86, 1.19)	0.885	1.01 (10.00, 1.02)	0.371
Cerebrovascular diseases	0.90 (0.79, 1.01)	0.073	0.89 (0.74, 1.07)	0.215
Alzheimer’s disease	0.98 (0.85, 1.14)	0.824	1.08 (0.93, 1.26)	0.298
Diabetes mellitus	0.89 (0.75, 1.04)	0.143	0.82 (0.65, 1.04)	0.101
Influenza and pneumonia	0.71 (0.57, 0.88)	**0.002**	0.71 (0.55, 0.93)	**0.012**
Nephritis, nephrotic syndrome and nephrosis	0.76 (0.62, 0.93)	**0.007**	0.74 (0.53, 1.03)	0.076
All other causes	0.79 (0.74, 0.84)	**< 0.001**	0.79 (0.73, 0.86)	**< 0.001**

Note: 25(OH)D was analyzed as a standardized continuous variable. HRs represent the change in mortality risk per one-SD increase in 25(OH)D levels.

Abbreviations: HR = Hazard ratios; CI = Confidence interval; SD = Standard deviation; 25(OH)D = 25-hydroxyvitamin D. HRs were adjusted for age, sex, race, annual household income, marital status, education level, BMI, diabetes, hypertension, weak/failing kidneys, and total cholesterol.

The multivariable adjusted model showed a strong inverse correlation between vitamin D status and all-cause mortality. Compared to the vitamin D deficiency group, the HR for all-cause mortality in the insufficiency group was 0.71 (weighted, 0.66 to 0.76), and in the sufficiency group, it was 0.64 (weighted, 95% CI 0.58 to 0.70). We further investigated the relationship between vitamin D status and cause-specific mortality (Table 3). The results showed a negative correlation between vitamin D status and certain cause-specific mortalities. Compared to the vitamin D deficiency group, the HR for cause-diseases of heart mortality in the insufficiency group was 0.66 (weighted, 0.56 to 0.76), for cause-malignant neoplasms mortality it was 0.66 (weighted, 0.55 to 0.79), for cause-chronic lower respiratory diseases it was 0.71 (weighted, 0.55 to 0.91), for cause-nephritis, nephrotic syndrome and nephrosis mortality it was 0.61 (weighted, 0.38 to 0.99), and for cause-all other causes mortality it was 0.73 (weighted, 0.62 to 0.86). Compared to the vitamin D deficiency group, the HR for cause-diseases of heart mortality in the sufficiency group was 0.64 (weighted, 0.58 to 0.70), for cause-malignant neoplasms mortality it was 0.61 (weighted, 0.51 to 0.81), for cause-chronic lower respiratory diseases mortality it was 0.63 (weighted, 0.45 to 0.90), for cause-influenza and pneumonia mortality it was 0.48 (weighted, 0.26 to 0.88), for cause-nephritis, nephrotic syndrome and nephrosis mortality it was 0.51 (weighted, 0.28 to 0.94), and for cause-all other causes mortality it was 0.57 (weighted, 0.48 to 0.68). In contrast, there was no significant correlation between vitamin D status and cause-specific mortalities from accidents, cerebrovascular diseases, Alzheimer’s disease, or diabetes.

**Table 3:** HR (95% CI) of all-cause mortality and cause specific mortality according to vitamin D status.

	Unweighted		Weighted	
Underlying Leading Cause of Death	HR (95% CI)	p-value	HR (95% CI)	p-value
all-cause mortality				
Deficiency (25(OH)D < 50 nmol/L)	reference		reference	
Insufficiency (50 nmol/L ≤ 25(OH)D ≤ 75 nmol/L)	0.73 (0.69, 0.77)	**<0.001**	0.71 (0.66, 0.76)	**<0.001**
Sufficiency (25(OH)D > 75 nmol/L)	0.67 (0.63, 0.72)	**<0.001**	0.64 (0.58, 0.70)	**<0.001**
Cause specific mortality				
Diseases of heart				
Deficiency (25(OH)D < 50 nmol/L)	reference		reference	
Insufficiency (50 nmol/L ≤ 25(OH)D ≤ 75 nmol/L)	0.70 (0.62, 0.79)	**<0.001**	0.66 (0.56, 0.76)	**<0.001**
Sufficiency (25(OH)D > 75 nmol/L)	0.66 (0.58, 0.75)	**<0.001**	0.61 (0.51, 0.73)	**<0.001**
Malignant neoplasms				
Deficiency (25(OH)D < 50 nmol/L)	reference		reference	
Insufficiency (50 nmol/L ≤ 25(OH)D ≤ 75 nmol/L)	0.67 (0.59, 0.77)	**<0.001**	0.66 (0.55, 0.79)	**<0.001**
Sufficiency (25(OH)D > 75 nmol/L)	0.74 (0.64, 0.85)	**<0.001**	0.67 (0.55, 0.81)	**<0.001**
Chronic lower respiratory diseases				
Deficiency (25(OH)D < 50 nmol/L)	reference		reference	
Insufficiency (50 nmol/L ≤ 25(OH)D ≤ 75 nmol/L)	0.77 (0.59, 10.00)	**0.048**	0.71 (0.55, 0.91)	**0.008**
Sufficiency (25(OH)D > 75 nmol/L)	0.68 (0.51, 0.91)	**0.01**	0.63 (0.45, 0.90)	**0.01**
Accidents				
Deficiency (25(OH)D < 50 nmol/L)	reference		reference	
Insufficiency (50 nmol/L ≤ 25(OH)D ≤ 75 nmol/L)	0.84 (0.59, 1.20)	0.347	0.87 (0.58, 1.31)	0.513
Sufficiency (25(OH)D > 75 nmol/L)	0.90 (0.61, 1.31)	0.574	0.96 (0.62, 1.50)	0.869
Cerebrovascular diseases				
Deficiency (25(OH)D < 50 nmol/L)	reference		reference	
Insufficiency (50 nmol/L ≤ 25(OH)D ≤ 75 nmol/L)	0.82 (0.63, 1.05)	0.114	0.75 (0.52, 1.08)	0.117
Sufficiency (25(OH)D > 75 nmol/L)	0.76 (0.57, 1.01)	0.059	0.70 (0.48, 1.02)	0.062
Alzheimer’s disease				
Deficiency (25(OH)D < 50 nmol/L)	reference		reference	
Insufficiency (50 nmol/L ≤ 25(OH)D ≤ 75 nmol/L)	0.88 (0.64, 1.21)	0.431	1.01 (0.72, 1.42)	0.962
Sufficiency (25(OH)D > 75 nmol/L)	0.90 (0.63, 1.28)	0.565	1.14 (0.75, 1.72)	0.537
Diabetes mellitus				
Deficiency (25(OH)D < 50 nmol/L)	reference		reference	
Insufficiency (50 nmol/L ≤ 25(OH)D ≤ 75 nmol/L)	0.73 (0.53, 10.00)	0.053	0.74 (0.48, 1.15)	0.177
Sufficiency (25(OH)D > 75 nmol/L)	0.72 (0.50, 1.05)	0.092	0.63 (0.38, 1.06)	0.081
Influenza and pneumonia				
Deficiency (25(OH)D < 50 nmol/L)	reference		reference	
Insufficiency (50 nmol/L ≤ 25(OH)D ≤ 75 nmol/L)	0.63 (0.42, 0.95)	**0.027**	0.74 (0.46, 1.19)	0.211
Sufficiency (25(OH)D > 75 nmol/L)	0.47 (0.29, 0.76)	**0.002**	0.48 (0.26, 0.88)	**0.017**
Nephritis, nephrotic syndrome and nephrosis				
Deficiency (25(OH)D < 50 nmol/L)	reference		reference	
Insufficiency (50 nmol/L ≤ 25(OH)D ≤ 75 nmol/L)	0.55 (0.36, 0.83)	**0.005**	0.61 (0.38, 0.99)	**0.047**
Sufficiency (25(OH)D > 75 nmol/L)	0.56 (0.36, 0.88)	**0.012**	0.51 (0.28, 0.94)	**0.03**
All other causes				
Deficiency (25(OH)D < 50 nmol/L)	reference		reference	
Insufficiency (50 nmol/L ≤ 25(OH)D ≤ 75 nmol/L)	0.77 (0.68, 0.86)	**<0.001**	0.73 (0.62, 0.86)	**<0.001**
Sufficiency (25(OH)D > 75 nmol/L)	0.59 (0.51, 0.67)	**<0.001**	0.57 (0.48, 0.68)	**<0.001**

Abbreviations: HR = Hazard ratios; CI = Confidence interval; SD = Standard deviation; 25(OH)D = 25-hydroxyvitamin D. HRs were adjusted for age, sex, race, annual household income, marital status, education level, BMI, diabetes, hypertension, weak/failing kidneys, and total cholesterol.

We conducted subgroup analyses based on selected characteristics (Table 4). The HR per SD for males was 0.84 (weighted, 0.79 to 0.90, SD = 23.71 nmol/L), while for females, it was 0.82 (weighted, 0.77 to 0.87, SD = 30.01 nmol/L). No significant correlation was found among individuals aged 18–39. The HR per SD was 0.74 (weighted, 0.65 to 0.85, SD = 26.22 nmol/L) among individuals aged 40–59. Among individuals aged 60 years or older, the HR per SD was 0.86 (weighted, 0.82 to 0.90, SD = 30.4 nmol/L).

**Table 4:** HR (95% CI) of all-cause mortality according to 25(OH)D levels in different sex and age groups.

Characteristic	Unweighted			Weighted		
	HR per SD (95% CI)	SD	p-value	HR per SD (95% CI)	SD	p-value
Sex						
Male	0.84 (0.80, 0.87)	23.98	**<0.001**	0.84 (0.79, 0.90)	23.71	**<0.001**
Female	0.84 (0.80, 0.87)	29.05	**<0.001**	0.82 (0.77, 0.87)	30.01	**<0.001**
Age						
18-39	0.89 (0.75, 1.05)	24.14	0.162	0.86 (0.68, 1.07)	24.87	0.177
40-59	0.74 (0.68, 0.81)	25.28	**<0.001**	0.74 (0.65, 0.85)	26.22	**<0.001**
60 and over	0.85 (0.83, 0.88)	29.63	**<0.001**	0.86 (0.82, 0.90)	30.4	**<0.001**

Note: 25(OH)D was analyzed as a standardized continuous variable. HRs represent the change in mortality risk per one-SD increase in 25(OH)D levels.

Abbreviations: HR = Hazard ratios; CI = Confidence interval; SD = Standard deviation; 25(OH)D = 25-hydroxyvitamin D. HRs were adjusted for age, sex, race, annual household income, marital status, education level, BMI, diabetes, hypertension, weak/failing kidneys, and total cholesterol.

Stratified analyses examining the relationship between vitamin D status and mortality across various demographic characteristics are detailed in S5 Table. Gender-specific analyses revealed distinct mortality patterns: among males, vitamin D insufficiency and sufficiency groups demonstrated reduced mortality risk compared to the deficiency group, with weighted HR of 0.73 (95% CI: 0.65–0.81) and 0.67 (95% CI: 0.58–0.76), respectively. Parallel findings were observed in females, where the weighted HRs were 0.70 (95% CI: 0.63–0.77) for insufficiency and 0.61 (95% CI: 0.55–0.69) for sufficiency groups, relative to deficiency.

Age-stratified analyses yielded heterogeneous associations. While no significant correlation emerged between vitamin D status and all-cause mortality in the 18−39 age group, substantial protective associations were observed in older cohorts. Among individuals aged 40−59 years, vitamin D insufficiency and sufficiency groups exhibited lower mortality risks compared to the deficiency group, with weighted HRs of 0.61 (95% CI: 0.50–0.73) and 0.55 (95% CI: 0.44–0.70), respectively. Similarly, in participants aged ≥60 years, the weighted HRs were 0.73 (95% CI: 0.67–0.80) for insufficiency and 0.67 (95% CI: 0.61–0.74) for sufficiency groups.

Notably, despite the absence of association with all-cause mortality in the youngest age group (18−39 years), cause-specific mortality analysis revealed a significant protective effect against malignant neoplasm-related mortality in the vitamin D sufficiency group (weighted HR: 0.17, 95% CI: 0.04–0.74) compared to the deficiency group, as documented in S6 Table.

### RCS with cox regression

**Fig 4:**
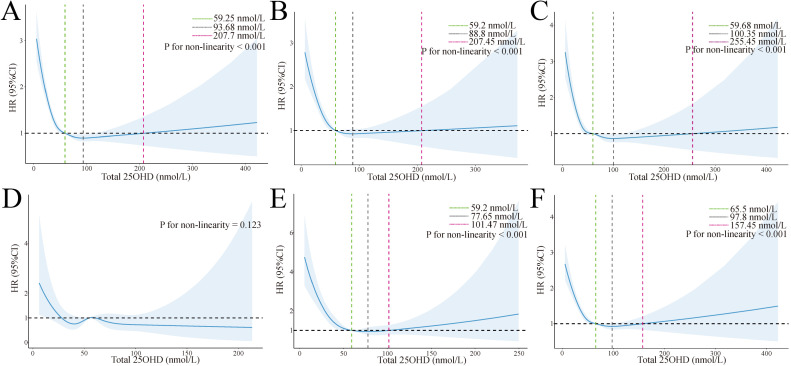
Association of total 25(OH)D levels with all-cause mortality, weigted. Association of total 25(OH)D levels with all-cause mortality in all individuals (A), female individuals (B), male individuals (C), individuals aged 18-39 (D), individuals aged 40-59 (E), and individuals aged 60 or older (F). Hazard ratios are indicated by solid lines and 95% CIs by shaded areas. Knots placed at 5th, 27.5th, 50th, 72.5th, and 95th centiles of 25(OH)D distribution. All models were adjusted for age, sex, race, annual household income, marital status, education level, BMI, diabetes, hypertension, weak/failing kidneys, and total cholesterol. Abbreviations: HR = Hazard ratios; CI = Confidence interval; 25(OH)D = 25-hydroxyvitamin D.

The RCS analysis revealed a non-linear relationship between 25(OH)D levels and mortality As depicted in Fig 4A, within the 25(OH)D range of 59.25 to 261.45 nmol/L, the HR decreased significantly, reaching its lowest HR at 96.81 nmol/L (weighted, non-linear p < 0.001). The unweighted result is presented in S8 Fig, indicating a HR reduction within the 25(OH)D range of 59.25 to 207.7 nmol/L, reaching the lowest HR at 93.68 nmol/L.

Furthermore, we conducted subgroup analyses based on selected factors. Fig 4B demonstrates that HRs were consistently below 1 for 25(OH)D levels above 59.2 nmol/L (weighted). Fig 4C presents that, for females, within the 25(OH)D range of 59.68 to 375.12 nmol/L, the HR decreased significantly, reaching the lowest HR at 102.9 nmol/L (weighted). Fig 4D shows that no significant non-linear relationship was observed between 25(OH)D levels and mortality among individuals aged 18–39 (non-linear p = 0.575). Fig 4E presents that, for individuals aged 18–39, within the range of 25(OH)D levels from 59.2 to 81.45 nmol/L, the HR decreased significantly, reaching the lowest risk at 67 nmol/L (weighted). Fig 4F presents that, for individuals aged 60 or older, within the 25(OH)D range of 65.5 to 225.43 nmol/L, the HR decreased significantly, reaching the HR risk at 104.23 nmol/L (weighted).

## Discussion

Our study reveals three key findings: First, 25(OH)D levels and vitamin D status were inversely associated with all-cause mortality, with the lowest mortality risk observed at 25(OH)D levels of 67–104 nmol/L across age groups. Second, the strongest association between vitamin D and reduced mortality was observed among individuals aged 40–59 years. Third, this inverse association was particularly notable for specific causes of death, including influenza and pneumonia, heart diseases, and malignant neoplasms.

While traditional vitamin D sufficiency thresholds (>50 nmol/L) were established based primarily on bone health outcomes, our RCS analyses suggest that optimal levels for mortality outcomes may be higher. Our observed optimal range of 67–104 nmol/L is remarkably consistent with previous studies. Bischoff-Ferrari et al. (2006) found that the most advantageous serum concentrations of 25(OH)D begin at 75 nmol/L, with optimal levels between 90–100 nmol/L for multiple health outcomes including bone mineral density, lower-extremity function, and fracture risk [11]. This was further confirmed in their 2014 review, which demonstrated that levels close to 75 nmol/L appeared most beneficial across various health endpoints [12]. More recently, Grant (2022) reported that optimal 25(OH)D concentrations above 75 nmol/L were associated with better outcomes for cardiovascular disease and all-cause mortality [13]. The consistency between our findings and these previous studies provides additional support for the robustness of this optimal range, suggesting that mortality benefits may require higher vitamin D than the traditional sufficiency threshold of 50 nmol/L.

Compared to previous studies, our research included a broader range of ages and ethnicities. Our findings are further supported by recent clinical guidelines. The Endocrine Society’s 2024 Clinical Practice Guideline on Vitamin D for the Prevention of Disease recommends vitamin D supplementation for adults aged 75 years and older to reduce mortality risk [14]. This recommendation aligns closely with our observed protective associations in the ≥ 60 years age group, where vitamin D insufficiency and sufficiency groups demonstrated significantly lower mortality risks compared to the deficiency group (HR: 0.73, 95% CI: 0.67–0.80 for insufficiency; HR: 0.67, 95% CI: 0.61–0.74 for sufficiency). The consistency between our population-based findings and these evidence-based clinical guidelines strengthens the clinical relevance of maintaining adequate vitamin D in older adults for mortality reduction. For the first time, our study revealed that no association between 25(OH)D levels and mortality among individuals aged 18–39. Unexpectedly, individuals aged 40–59 exhibited a stronger negative correlation between 25(OH)D levels and mortality compared to other age groups, suggesting that vitamin D supplementation may have greater advantages for this specific age range. Furthermore, RCS analysis revealed a J-shaped relationship between all-cause mortality and 25(OH)D. In the 18–39 age group, no non-linear relationship was observed. The lowest mortalities were observed when 25(OH)D levels were around 67–104 nmol/L.

The mechanistic pathways through which vitamin D influences mortality are multifaceted. The bioactive form of vitamin D [1,25(OH)₂D₃] interacts with its receptor (VDR) to regulate gene expression across various tissues [2,7,10,15]. This ligand-receptor complex orchestrates the tissue- and cell-specific modulation of gene expression patterns, thereby exerting precise control over cellular phenotypes and physiological functions.Experimental investigations have established that 1,25(OH)2D3 demonstrates dual capacity in cellular regulation, simultaneously inhibiting proliferation and promoting terminal differentiation in both normal and neoplastic cell populations. A principal molecular mechanism underlying these effects involves the suppression of epithelial-mesenchymal transition through antagonistic interactions with multiple signaling cascades, including Wnt/β-catenin, TGF-β, and EGF pathways [16–19]. Furthermore, vitamin D exhibits direct regulatory control over cell cycle progression through modulation of cell cycle checkpoint proteins, culminating in cell cycle arrest and subsequent reduction in neoplastic cell proliferation [20].

The anticancer properties attributed to vitamin D extend beyond direct cellular regulation to encompass immunomodulatory functions, particularly in the context of immune cell growth and differentiation processes [7]. This multifaceted regulatory network underscores the pivotal role of vitamin D in maintaining cellular homeostasis and preventing pathological processes.

It is common to find vitamin D deficiency in respiratory system diseases, and this deficiency is linked to heightened disease severity [21–23]. vitamin D can regulate the ongoing abnormal immune response in chronic respiratory system diseases and limit the colonization of bacteria and viruses in the lungs [22]. Previous studies have shown that vitamin D may reduce inflammation caused by T cells [24,25]. Further studies have found that in the lung cells of COVID-19 patients, certain immune responses may enter an exaggerated state, exacerbating the inflammatory manifestations in the lungs. Complement triggers contraction of the T-helper (Th) 1 cell response by inducing intrinsic expression of VDR and the vitamin D activating enzyme CYP27B1, allowing T cell activation and response to vitamin D. Subsequently, vitamin D initiates the transition of T cells from a pro-inflammatory state to an anti-inflammatory state. In theory, vitamin D may potentially help treat patients with severe inflammation caused by Th1 cells [26]. This may explain why 25(OH)D levels show the strongest negative correlation with specific mortality for influenza and pneumonia.

Vitamin D deficiency demonstrates significant associations with a spectrum of cardiovascular disorders, encompassing vascular dysfunction, atherosclerotic processes, left ventricular hypertrophy, hypertensive conditions, and dyslipidemic states [4,27–29]. These pathophysiological manifestations are mechanistically linked to VDR signaling through multiple molecular pathways. The cardiovascular effects of vitamin D are mediated through VDR activation in both myocardial and endothelial cellular compartments, while simultaneously modulating the renin-angiotensin-aldosterone system (RAAS), pancreatic cellular function, and metabolic energy homeostasis [30]. Experimental evidence derived from VDR-null murine models has provided compelling insights into the essential role of vitamin D signaling in cardiovascular homeostasis. These knockout models manifest a hypertensive phenotype characterized by enhanced RAAS activation. Moreover, the cardiac manifestations in VDR-deficient mice extend to significant myocardial hypertrophy, quantitatively demonstrated by elevated heart weight-to-body weight ratios and augmented expression profiles of natriuretic peptides [31,32].

In the context of CKD, severe vitamin D deficiency occurs due to dietary restrictions in patients and reduced sun exposure resulting from complications that may affect hospitalization and physical activity. This severe deficiency further leads to CKD-related mineral and bone disorders (CKD-MBD). CKD-MBD complications encompass a range of adverse outcomes, such as stroke, vascular calcification, skeletal fractures, and an elevated susceptibility to mortality [33]. In both mouse models of acute kidney injury (AKI) and renal tissues from AKI patients, there is a persistent downregulation of VDR expression [34]. Knocking out VDR can exacerbate renal injury and cell apoptosis in a mouse model of AKI, while VDR agonist paricalcitol and VDR overexpression can alleviate this through the ATF4/CHOP pathway and inhibition of oxidative stress [34,35].

Our study is the first population-based investigation of the association between 25(OH)D levels, vitamin D status, and mortality. Compared to other studies, our research is based on the NHANES database, which utilizes a complex sampling design and includes individuals from a wider age range and diverse ethnic backgrounds. This ensures participant diversity and yields conclusions with higher external validity. This study incorporates multiple covariates that may influence 25(OH)D levels, thereby minimizing confounding factors and assuring us of the reliability of the statistical results.

Several limitations of our study should be acknowledged. First, despite comprehensive adjustment for multiple covariates in our analyses, the observational nature of this study limits our ability to establish causality between vitamin D and mortality outcomes. The observed associations may be influenced by residual confounding factors not captured in our analyses, and reverse causation cannot be completely ruled out, as underlying health conditions might affect both vitamin D and mortality risk.

Second, a critical limitation is our inability to adjust for several key lifestyle and environmental factors that significantly influence vitamin D metabolism and status. Sunlight exposure, the primary determinant of vitamin D synthesis, could not be adequately controlled for in our analyses. While NHANES collected outdoor activity data during 2009–2018, this information does not directly represent UVB exposure, as effective vitamin D synthesis depends on geographical latitude, season, time of day, weather conditions, skin pigmentation, and sunscreen use. Vitamin D supplementation represents another major unmeasured variable, as comprehensive supplement data were not consistently available across all NHANES cycles, preventing us from distinguishing between individuals achieving adequate 25(OH)D levels through natural synthesis versus supplementation. This limitation is particularly important given that supplement users may differ systematically from non-users in health consciousness and healthcare behaviors. Seasonal variations in 25(OH)D levels, which can fluctuate by 15–20 nmol/L throughout the year, could not be fully accounted for despite our multi-year data collection. Additionally, genetic polymorphisms affecting vitamin D metabolism, including variants in genes encoding vitamin D binding protein, 25-hydroxylase, and vitamin D receptor, were not available in our dataset and may modify individual responses to vitamin D exposure.

Third, although NHANES is a long-term tracking survey, each data collection round is cross-sectional, providing 25(OH)D measurements only at specific time points and unable to capture long-term changes in vitamin D status within individuals. This single-measurement approach may not reflect individuals’ usual vitamin D status over the follow-up period. Fourth, the methods of data collection, including questionnaire surveys, physical measurements, and biological samples, may be subject to recall bias and measurement errors, potentially affecting the accuracy and reliability of the data. Finally, our study results suggest an optimal threshold for 25(OH)D levels around 67–104 nmol/L. In our study, 7.9% of individuals had 25(OH)D levels >100 nmol/L, which is much higher than the proportion reported in other study (1.5%) [36]. However, this proportion is still relatively low in the overall population, leading to wider CI in the RCS graphs as 25(OH)D levels increase. Therefore, caution should be exercised when extrapolating our study results to higher levels.

## Conclusions

Higher 25(OH)D levels were associated with lower all-cause mortality risk, with the lowest mortality risk observed at 25(OH)D levels of 67–104 nmol/L (67 nmol/L for ages 40–59 and 104.23 nmol/L for ages ≥60 years). This association was strongest among individuals aged 40–59 years, suggesting age-specific benefits of vitamin D supplementation.

## Supporting information

S1 Fig Flow chart of participants enrolled into the study. (TIF)

S2 Fig Missing ratio of variables. Variables with missing proportions >5% were excluded from the final model. For remaining variables, missing values were imputed using the mice package in R, generating five imputed datasets. Results were pooled using Rubin’s rules, and sensitivity analyses comparing complete case analysis with imputed results were performed to assess robustness. Abbreviations: 25(OH)D = 25-hydroxyvitamin D; BMI = Body mass index.
(TIF)

S3 Fig Spearman correlation between variables and 25(OH)D level. (TIFF)

S4 Fig Cumulative hazard for all-cause mortality in all individuals (A), female individuals (B), male individuals (C), individuals aged 18–39 (D), individuals aged 40–59 (E), and individuals aged 60 or older (F). (TIF)

S5 Fig Kaplan-Meier survival probability and cumulative hazard for cause-specific mortalities. Kaplan-Meier survival probability and cumulative hazard for cause-chronic lower respiratory diseases (A), cause-accidents mortality (B), cause-cerebrovascular diseases mortality (C), and cause- Alzheimer’s disease mortality (D) in all individuals.
(TIF)

S6 Fig Kaplan-Meier survival probability and cumulative hazard for cause-specific mortalities. Kaplan-Meier survival probability and cumulative hazard for cause-diabetes mellitus mortality (A), cause-influenza and pneumonia mortality (B), and cause-nephritis, nephrotic syndrome and nephrosis mortality (C) in all individuals.
(TIF)

S7 Fig Hazard ratios (95% CI) of 25(OH)D and all covariates, unweigted. (TIF)

S8 Fig Association of total 25(OH)D levels with all-cause mortality, unweigted. Association of total 25(OH)D levels with all-cause mortality in all individuals (A), female individuals (B), male individuals (C), individuals aged 18–39 (D), individuals aged 40–59 (E), and individuals aged 60 or older (F). Hazard ratios are indicated by solid lines and 95% CIs by shaded areas. Knots placed at 5th, 27.5th, 50th, 72.5th, and 95th centiles of 25(OH)D distribution. All models were adjusted for age, sex, race, annual household income, marital status, education level, BMI, diabetes, hypertension, weak/failing kidneys, and total cholesterol.
(TIF)

S1 Table Cox Regression with stepwise variable reduction. (DOCX)

S2 Table Demographic characteristics of individuals according to status, weighted. (DOCX)

S3 Table Demographic characteristics of participants according to vitamin D status, unweighted. (DOCX)

S4 Table Demographic characteristics of participants according to status, unweighted. (DOCX)

S5 Table HR (95% CI) of all-cause mortality according to vitamin D status in different sex and age groups. (DOCX)

S6 Table HR (95% CI) of cause specific mortality according to vitamin D status in participants 18–39 years. (DOCX)
