# Supplementary figures and images for: Age-specific benefits of Vitamin D and its association with mortality
Source: PLoS One. 2025 Aug 29;20(8):e0330959. doi: 10.1371/journal.pone.0330959 (PMC12396682; doi:10.1371/journal.pone.0330959)

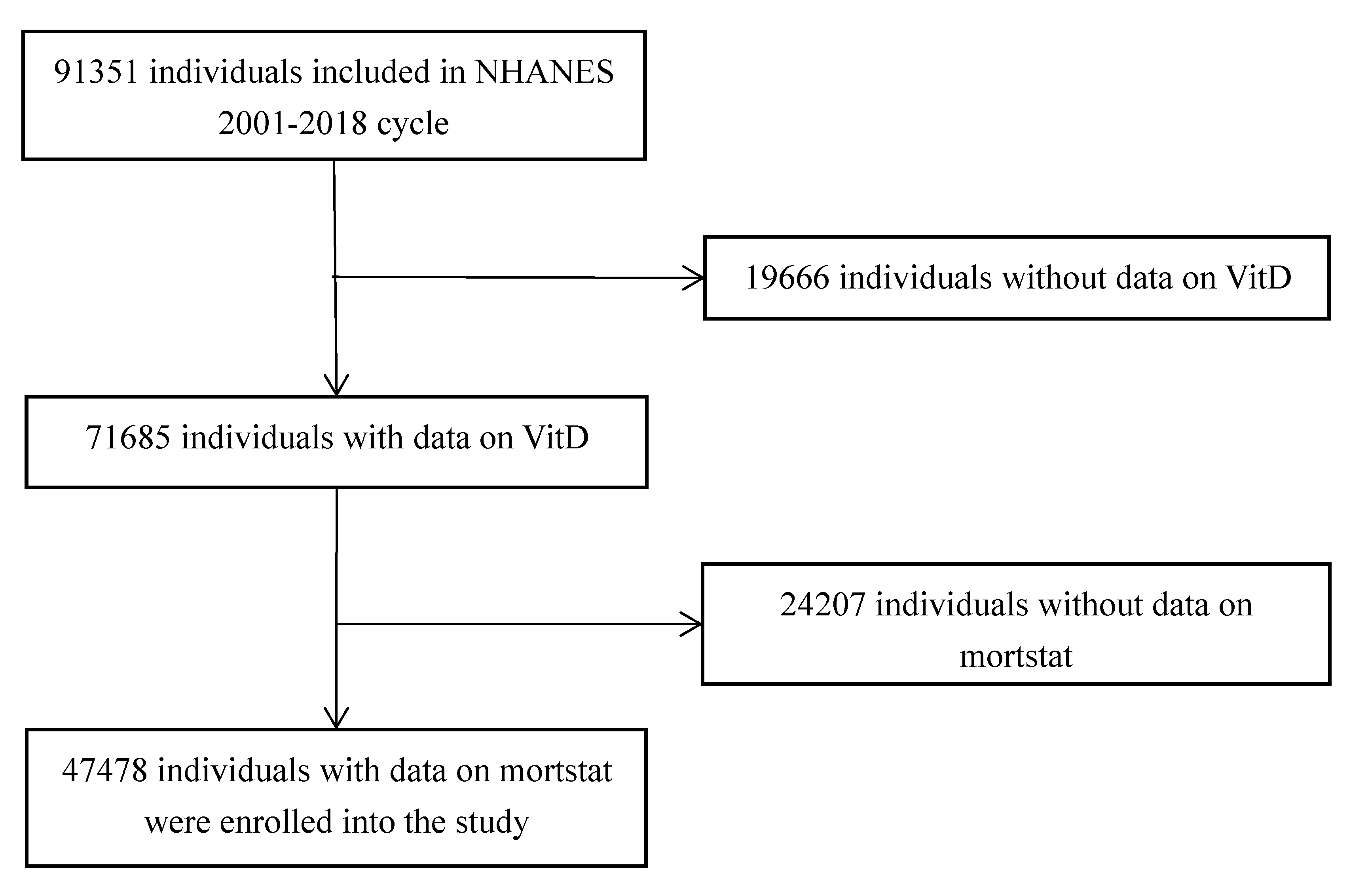

Supplement: S1 Fig — (TIF) [file pone.0330959.s001.tif]

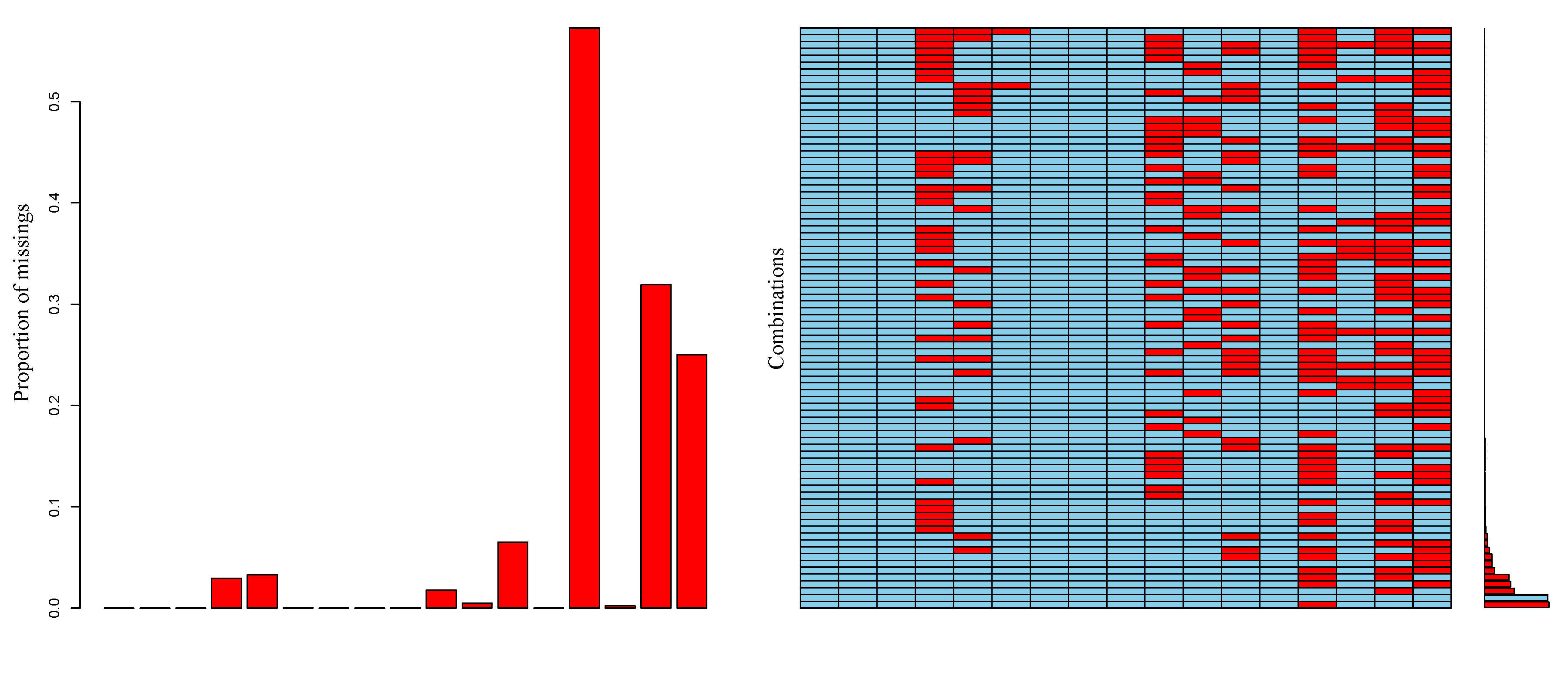

Supplement: S2 Fig — Variables with missing proportions >5% were excluded from the final model. For remaining variables, missing values were imputed using the mice package in R, generating five imputed datasets. Results were pooled using Rubin’s rules, and sensitivity analyses comparing complete case analysis with imputed results were performed to assess robustness. Abbreviations: 25(OH)D = 25-hydroxyvitamin D; BMI = Body mass index. (TIF) [file pone.0330959.s002.tif]

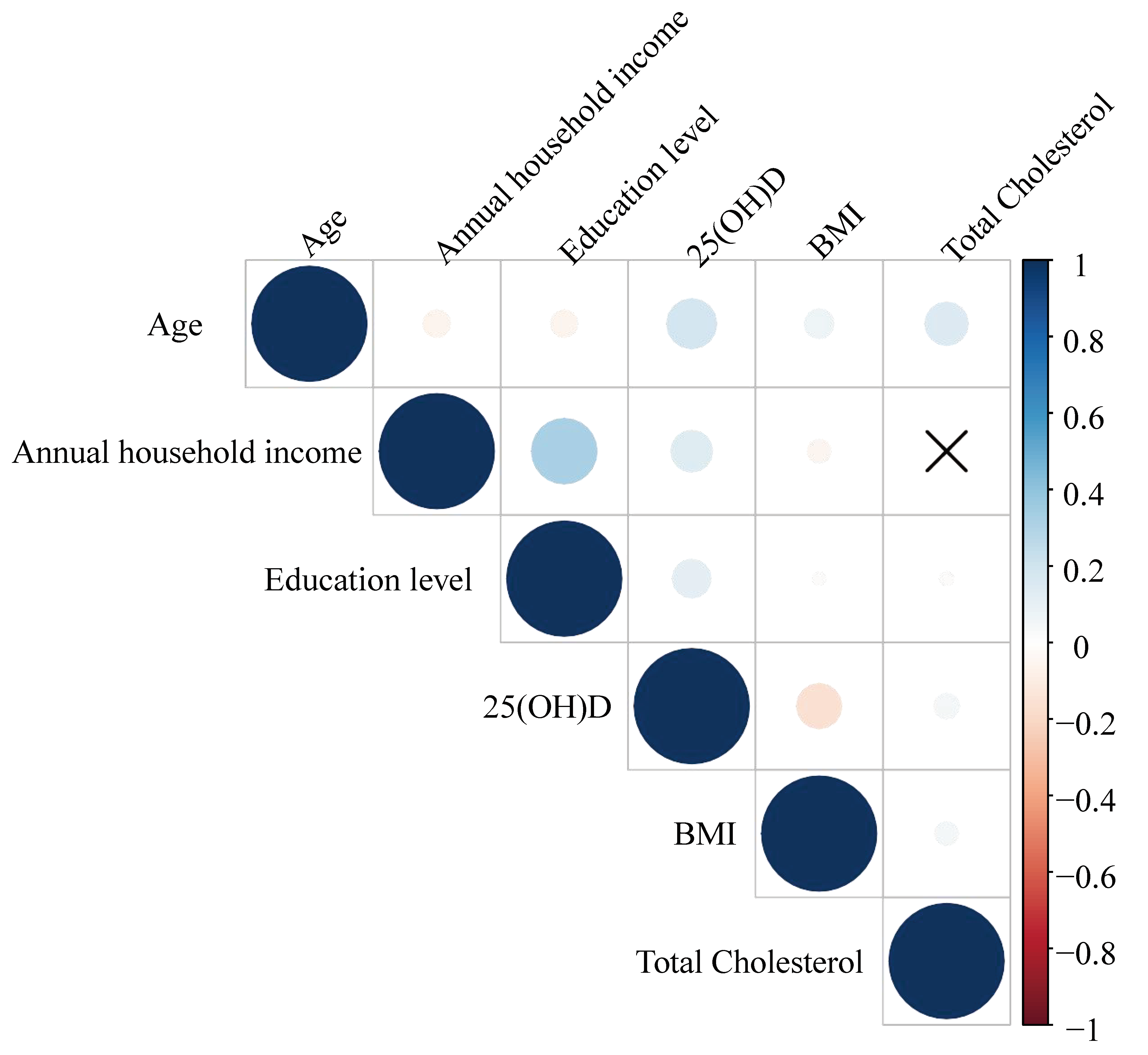

Supplement: S3 Fig — (TIFF) [file pone.0330959.s003.tiff]

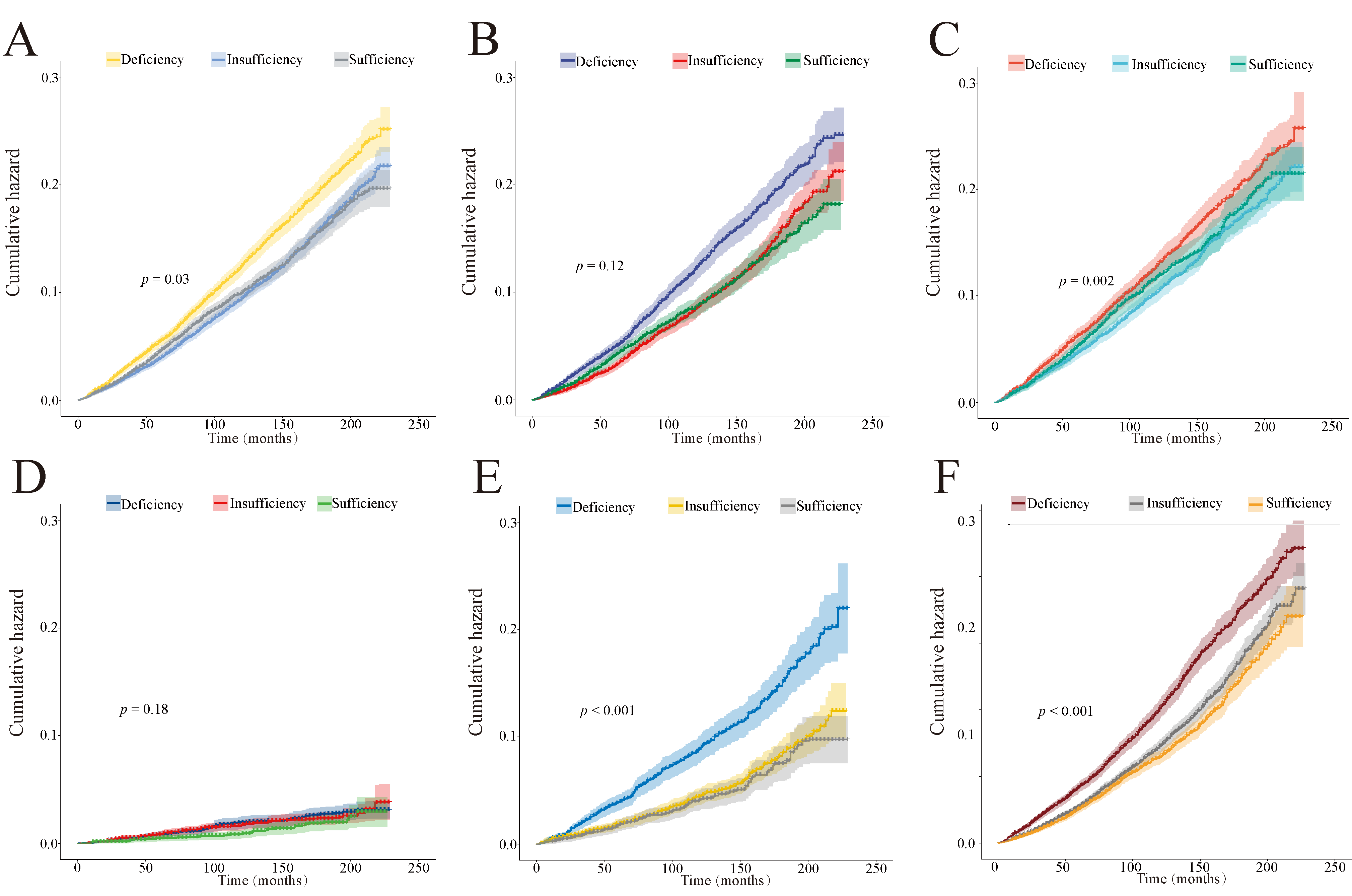

Supplement: S4 Fig — (TIF) [file pone.0330959.s004.tif]

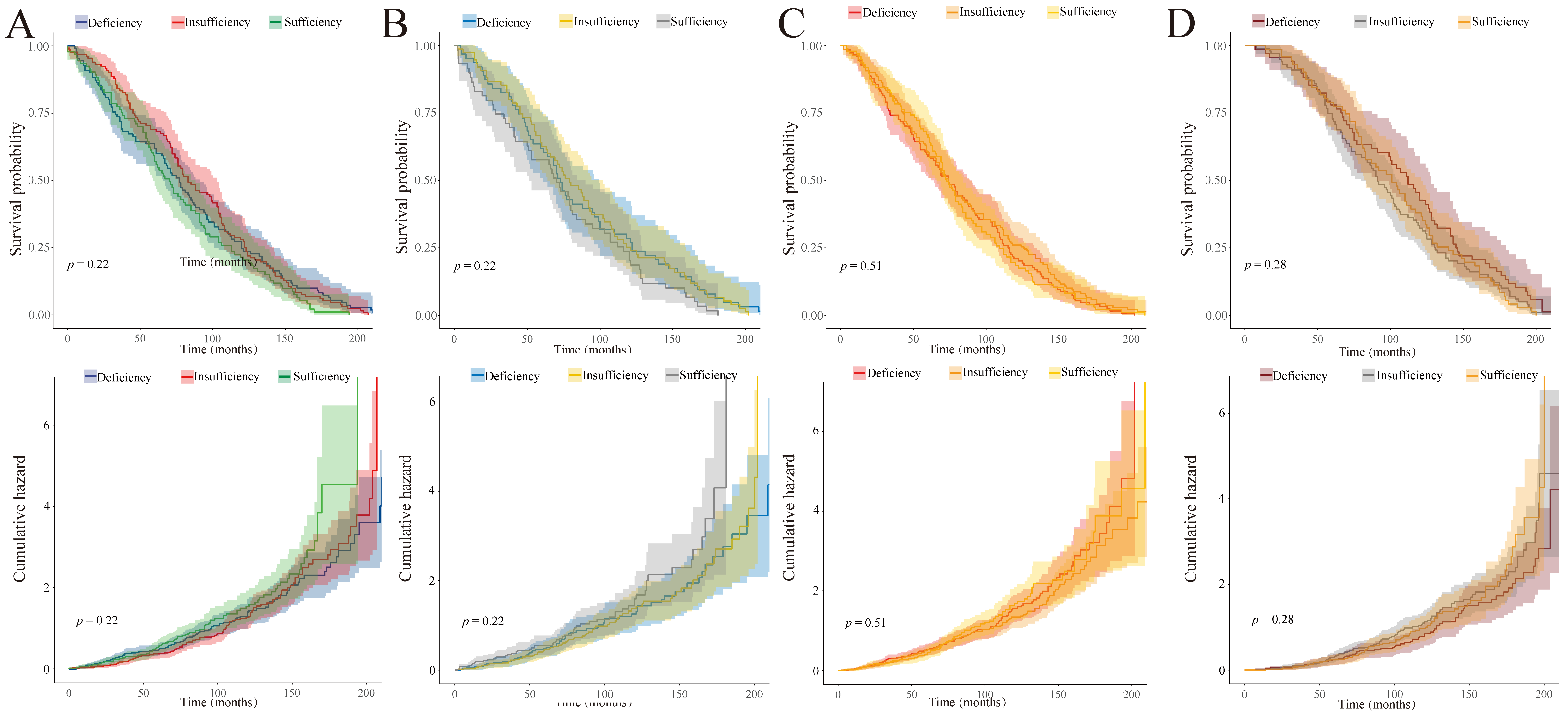

Supplement: S5 Fig — Kaplan-Meier survival probability and cumulative hazard for cause-chronic lower respiratory diseases (A), cause-accidents mortality (B), cause-cerebrovascular diseases mortality (C), and cause- Alzheimer’s disease mortality (D) in all individuals. (TIF) [file pone.0330959.s005.tif]

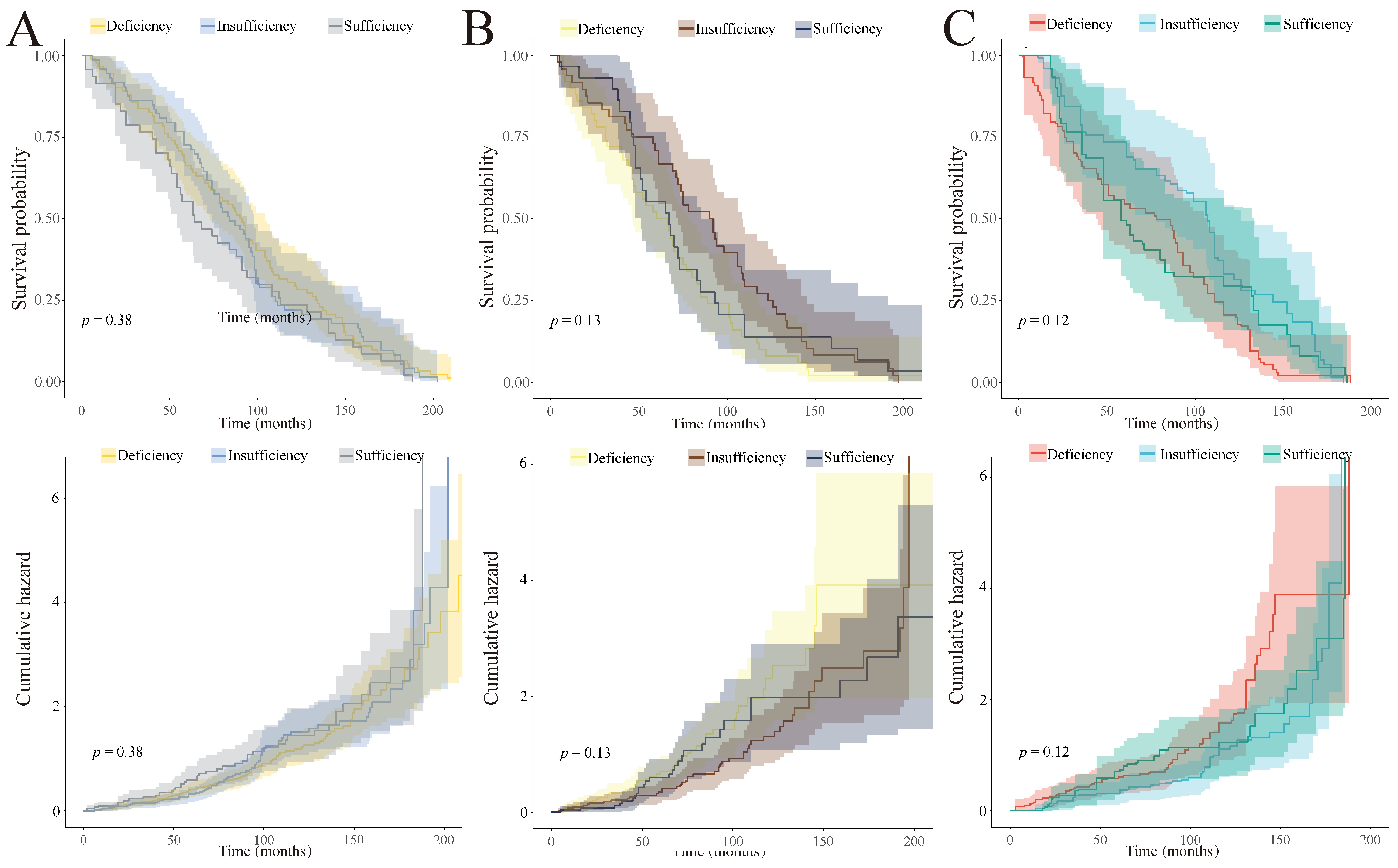

Supplement: S6 Fig — Kaplan-Meier survival probability and cumulative hazard for cause-diabetes mellitus mortality (A), cause-influenza and pneumonia mortality (B), and cause-nephritis, nephrotic syndrome and nephrosis mortality (C) in all individuals. (TIF) [file pone.0330959.s006.tif]

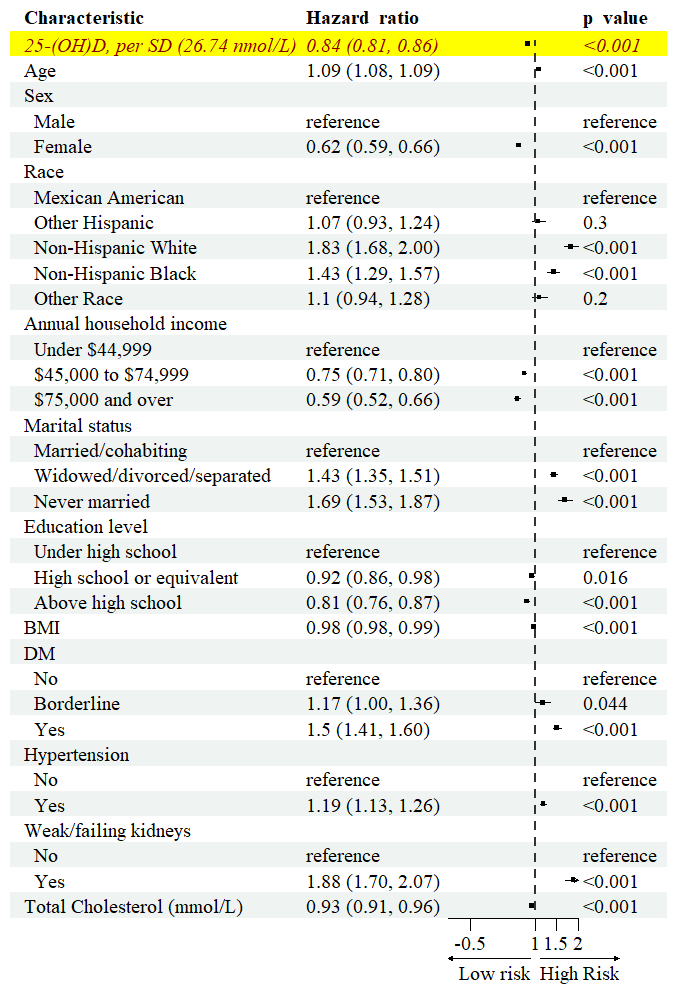

Supplement: S7 Fig — (TIF) [file pone.0330959.s007.tif]

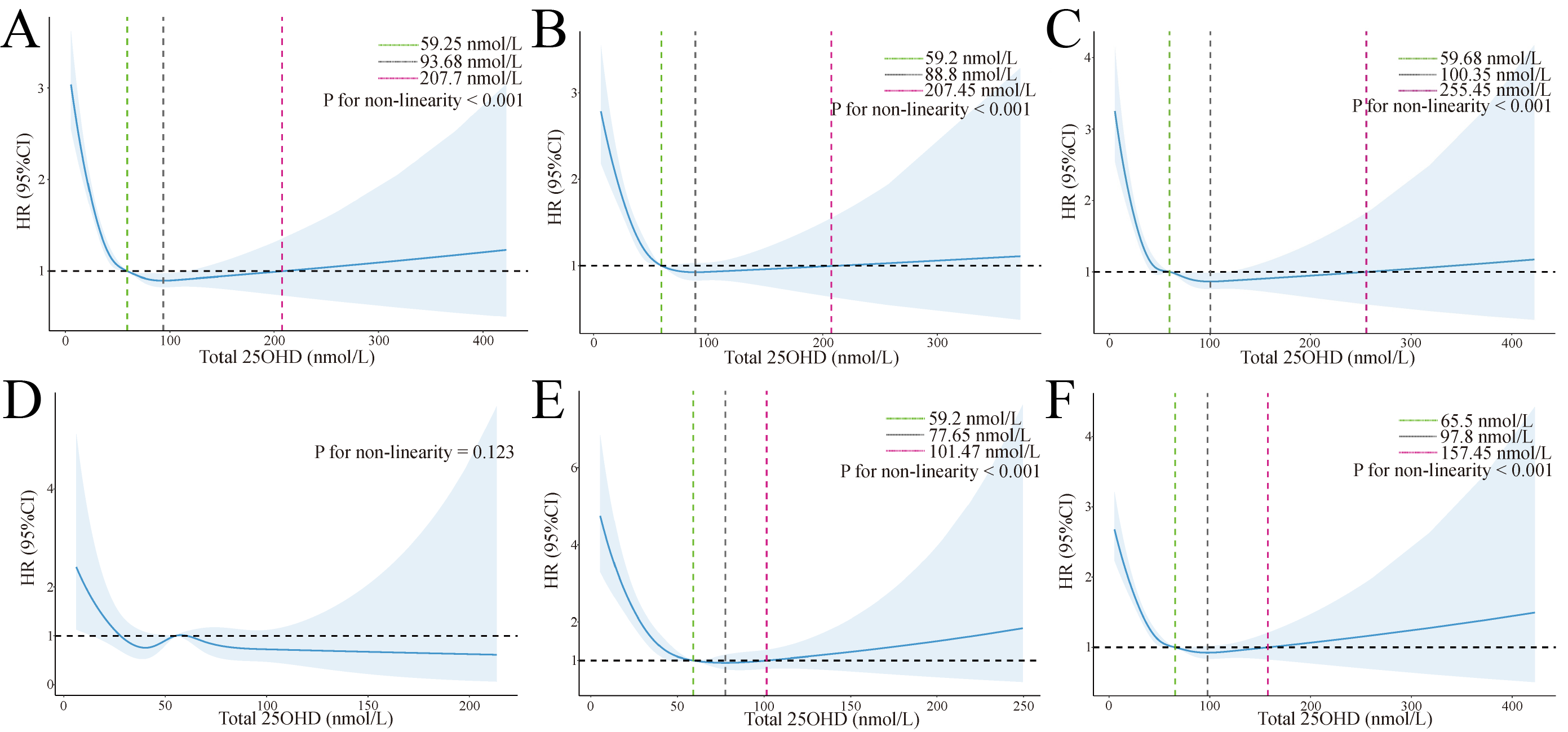

Supplement: S8 Fig — Association of total 25(OH)D levels with all-cause mortality in all individuals (A), female individuals (B), male individuals (C), individuals aged 18–39 (D), individuals aged 40–59 (E), and individuals aged 60 or older (F). Hazard ratios are indicated by solid lines and 95% CIs by shaded areas. Knots placed at 5th, 27.5th, 50th, 72.5th, and 95th centiles of 25(OH)D distribution. All models were adjusted for age, sex, race, annual household income, marital status, education level, BMI, diabetes, hypertension, weak/failing kidneys, and total cholesterol. (TIF) [file pone.0330959.s008.tif]
