# Supplementary material for: Age-specific benefits of Vitamin D and its association with mortality
Source: PLoS One. 2025 Aug 29;20(8):e0330959. doi: 10.1371/journal.pone.0330959 (PMC12396682; doi:10.1371/journal.pone.0330959)
Supplement: S1 Table — (DOCX) [file pone.0330959.s009.docx]

| **Characteristic** |  | HR (univariable) | HR (multivariable) |
| --- | --- | --- | --- |
| Age |  | 1.09 (1.09-1.10, p<0.001) | 1.09 (1.08-1.09, p<0.001) |
| Race |  |  |  |
|  | Other Race | reference | reference |
|  | Mexican American | 1.10 (0.95-1.29, p=0.205) | 0.91 (0.78-1.06, p=0.238) |
|  | Other Hispanic | 1.19 (0.99-1.42, p=0.059) | 0.98 (0.82-1.17, p=0.811) |
|  | Non-Hispanic White | 2.73 (2.38-3.13, p<0.001) | 1.67 (1.45-1.92, p<0.001) |
|  | Non-Hispanic Black | 1.80 (1.55-2.08, p<0.001) | 1.30 (1.12-1.50, p=0.001) |
| Annual household income |  |  |  |
|  | Under $44,999 | reference | reference |
|  | $45,000 to $74,999 | 0.50 (0.47-0.53, p<0.001) | 0.75 (0.71-0.80, p<0.001) |
|  | $75,000 and over | 0.31 (0.28-0.35, p<0.001) | 0.59 (0.52-0.66, p<0.001) |
| Marital status |  |  |  |
|  | Married/cohabiting | reference | reference |
|  | Widowed/divorced/separated | 2.71 (2.58-2.86, p<0.001) | 1.43 (1.35-1.51, p<0.001) |
|  | Never married | 0.40 (0.36-0.44, p<0.001) | 1.69 (1.53-1.87, p<0.001) |
| Education level |  |  |  |
|  | Under high school | reference | reference |
|  | High school or equivalent | 0.77 (0.72-0.82, p<0.001) | 0.92 (0.86-0.98, p=0.016) |
|  | Above high school | 0.56 (0.53-0.60, p<0.001) | 0.81 (0.76-0.87, p<0.001) |
| 25(OH)D (nmol/L) |  | 10.00 (10.00-10.00, p=0.441) | 0.99 (0.99-0.99, p<0.001) |
| Sex |  |  |  |
|  | Men | reference | reference |
|  | Women | 0.71 (0.68-0.75, p<0.001) | 0.62 (0.59-0.66, p<0.001) |
| BMI | Mean (SD) | 10.00 (0.99-10.00, p=0.040) | 0.98 (0.98-0.99, p<0.001) |
| Diabetes |  |  |  |
|  | No | reference | reference |
|  | Borderline | 2.20 (1.89-2.56, p<0.001) | 1.17 (10.00-1.36, p=0.044) |
|  | Yes | 3.25 (3.06-3.44, p<0.001) | 1.50 (1.41-1.60, p<0.001) |
| Hypertension |  |  |  |
|  | No | reference | reference |
|  | Yes | 3.61 (3.43-3.80, p<0.001) | 1.19 (1.13-1.26, p<0.001) |
| Weak/failing kidneys |  |  |  |
|  | No | reference | reference |
|  | Yes | 3.64 (3.31-40.00, p<0.001) | 1.88 (1.70-2.07, p<0.001) |
| Total Cholesterol (mmol/L) |  | 0.97 (0.95-0.99, p=0.008) | 0.93 (0.91-0.96, p<0.001) |

Performance of multivaiate model: Number in dataframe = 47478, Number in model = 47478, Number of events = 6231, Concordance = 0.870 (SE = 0.002), R-squared = 0.250(Max possible = 0.931), Likelihood ratio test = 13653.320 (df = 19, p = 0.000)

Abbreviations: 25(OH)D = 25-hydroxyvitamin D; BMI = Body mass index.
