# Supplementary material for: Age-specific benefits of Vitamin D and its association with mortality
Source: PLoS One. 2025 Aug 29;20(8):e0330959. doi: 10.1371/journal.pone.0330959 (PMC12396682; doi:10.1371/journal.pone.0330959)
Supplement: S2 Table — (DOCX) [file pone.0330959.s010.docx]

| **Characteristic** | **Assumed alive** | **Assumed deceased** | **p-value** |
| --- | --- | --- | --- |
|  | **(N=191530035)** | **(N=20644280)** |  |
| Sex = female (%) | 99705982 (52.1) | 9999799 (48.4) | **< 0.001** |
| Age | 430.00 (30.00, 560.00) | 70.00 (580.00, 80.00) | **< 0.001** |
| Race (%) |  |  | **< 0.001** |
| Mexican American | 17313563 (9.0) | 760610 (3.7) |  |
| Other Hispanic | 10921767 (5.7) | 593936 (2.9) |  |
| Non-Hispanic White | 128125810 (66.9) | 16367425 (79.3) |  |
| Non-Hispanic Black | 20899955 (10.9) | 2145003 (10.4) |  |
| Other Race | 14268939 (7.4) | 777306 (3.8) |  |
| 25(OH)D (nmol/L) | 65.50 (49.50, 82.69) | 61.25 (44.40, 78.60) | **< 0.001** |
| Months of follow-up | 1060.00 (570.00, 1660.00) | 750.00 (380.00, 1210.00) | **< 0.001** |
| Annual household income (%) |  |  | **< 0.001** |
| Under $44,999 | 76080143 (39.7) | 13582072 (65.8) |  |
| $45,000 to $74,999 | 62521524 (32.6) | 5540686 (26.8) |  |
| $75,000 and over | 52928366 (27.6) | 1521522 (7.4) |  |
| Marital status (%) |  |  | **< 0.001** |
| Married/cohabiting | 122360373 (63.9) | 10896182 (52.8) |  |
| Widowed/divorced/separated | 30239025 (15.8) | 8057405 (39.0) |  |
| Never married | 38930635 (20.3) | 1690693 (8.2) |  |
| Education level (%) |  |  | **< 0.001** |
| Under high school | 30404066 (15.9) | 6048769 (29.3) |  |
| High school or equivalent | 45738215 (23.9) | 5734411 (27.8) |  |
| Above high school | 115387753 (60.2) | 8861100 (42.9) |  |
| BMI | 27.66 (240.00, 32.20) | 27.60 (24.01, 320.00) | 0.815 |
| Diabetes (%) |  |  | **< 0.001** |
| No | 173730407 (90.7) | 15582871 (75.5) |  |
| Borderline | 3348873 (1.7) | 587583 (2.8) |  |
| Yes | 14450753 (7.5) | 4473826 (21.7) |  |
| Hypertension = Yes (%) | 52262734 (27.3) | 11946099 (57.9) | **< 0.001** |
| Weak/failing kidneys = Yes (%) | 3755055 (2.0) | 1339188 (6.5) | **< 0.001** |
| Total Cholesterol (mmol/L) | 4.94 (4.27, 5.66) | 4.99 (4.24, 5.77) | .089 |
| Abbreviations: 25(OH)D = 25-hydroxyvitamin D; BMI = Body mass index. | | | |
