# Supplementary material for: Age-specific benefits of Vitamin D and its association with mortality
Source: PLoS One. 2025 Aug 29;20(8):e0330959. doi: 10.1371/journal.pone.0330959 (PMC12396682; doi:10.1371/journal.pone.0330959)
Supplement: S3 Table — (DOCX) [file pone.0330959.s011.docx]

| **Characteristic** | **Deficiency** | **Insufficiency** | **Sufficiency** | **p-value** |
| --- | --- | --- | --- | --- |
|  | **25(OH)D <50 nmol/L** | **50 nmol/L ≤25(OH)D ≤75 nmol/L** | **25(OH)D >75 nmol/L** |  |
|  | **(N=16666)** | **(N=17752)** | **(N=13060)** |  |
| Sex = female (%) | 8795 (52.8) | 8371 (47.2) | 7309 (56.0) | **<0.001** |
| Age | 420.00 (280.00, 59.75) | 460.00 (310.00, 620.00) | 540.00 (360.00, 690.00) | **<0.001** |
| Race (%) |  |  |  | **<0.001** |
| Mexican American | 3676 (22.1) | 3497 (19.7) | 1136 (8.7) |  |
| Other Hispanic | 1312 (7.9) | 1782 (10.0) | 848 (6.5) |  |
| Non-Hispanic White | 3665 (22.0) | 8326 (46.9) | 8686 (66.5) |  |
| Non-Hispanic Black | 6273 (37.6) | 2449 (13.8) | 1212 (9.3) |  |
| Other Race | 1740 (10.4) | 1698 (9.6) | 1178 (9.0) |  |
| 25(OH)D (nmol/L) | 37.45 (29.70, 44.24) | 61.60 (56.19, 680.00) | 89.35 (810.00, 102.87) | **<0.001** |
| Months of follow-up | 110.00 (60.00, 1650.00) | 1080.00 (590.00, 1620.00) | 930.00 (490.00, 1430.00) | **<0.001** |
| Annual household income (%) |  |  |  | **<0.001** |
| Under $44,999 | 10113 (60.7) | 9445 (53.2) | 6202 (47.5) |  |
| $45,000 to $74,999 | 4430 (26.6) | 5161 (29.1) | 3688 (28.2) |  |
| $75,000 and over | 2123 (12.7) | 3146 (17.7) | 3170 (24.3) |  |
| Marital status (%) |  |  |  | **<0.001** |
| Married/cohabiting | 8486 (50.9) | 10876 (61.3) | 8127 (62.2) |  |
| Widowed/divorced/separated | 3456 (20.7) | 3506 (19.7) | 2994 (22.9) |  |
| Never married | 4724 (28.3) | 3370 (19.0) | 1939 (14.8) |  |
| Education level (%) |  |  |  | **<0.001** |
| Under high school | 5241 (31.4) | 4948 (27.9) | 2599 (19.9) |  |
| High school or equivalent | 4057 (24.3) | 4159 (23.4) | 3127 (23.9) |  |
| Above high school | 7368 (44.2) | 8645 (48.7) | 7334 (56.2) |  |
| BMI | 28.82 (24.66, 33.99) | 27.70 (24.18, 31.90) | 26.60 (23.39, 30.54) | **<0.001** |
| Diabetes (%) |  |  |  | **<0.001** |
| No | 14315 (85.9) | 15518 (87.4) | 11157 (85.4) |  |
| Borderline | 321 (1.9) | 326 (1.8) | 305 (2.3) |  |
| Yes | 2030 (12.2) | 1908 (10.7) | 1598 (12.2) |  |
| Hypertension = Yes (%) | 5265 (31.6) | 5519 (31.1) | 4988 (38.2) | **<0.001** |
| Weak/failing kidneys = Yes (%) | 479 (2.9) | 430 (2.4) | 535 (4.1) | **<0.001** |
| Total Cholesterol (mmol/L) | 4.81 (4.16, 5.59) | 4.94 (4.24, 5.66) | 4.97 (4.27, 5.72) | **<0.001** |
| Abbreviations: 25(OH)D = 25-hydroxyvitamin D; BMI = Body mass index. | | | | |
