# Supplementary material for: Age-specific benefits of Vitamin D and its association with mortality
Source: PLoS One. 2025 Aug 29;20(8):e0330959. doi: 10.1371/journal.pone.0330959 (PMC12396682; doi:10.1371/journal.pone.0330959)
Supplement: S4 Table — (DOCX) [file pone.0330959.s012.docx]

| **Characteristic** | **Assumed alive** | **Assumed deceased** | **p-value** |
| --- | --- | --- | --- |
|  | **(N=41247)** | **(N=6231)** |  |
| Sex = female (%) | 21739 (52.7) | 2736 (43.9) | **<0.001** |
| Age | 430.00 (290.00, 590.00) | 730.00 (630.00, 80.00) | **<0.001** |
| Race (%) |  |  | **<0.001** |
| Mexican American | 7622 (18.5) | 687 (11.0) |  |
| Other Hispanic | 3678 (8.9) | 264 (4.2) |  |
| Non-Hispanic White | 16788 (40.7) | 3889 (62.4) |  |
| Non-Hispanic Black | 8759 (21.2) | 1175 (18.9) |  |
| Other Race | 4400 (10.7) | 216 (3.5) |  |
| 25(OH)D (nmol/L) | 59.45 (43.40, 77.15) | 58.70 (41.15, 75.40) | **<0.001** |
| Months of follow-up | 110.00 (60.00, 1640.00) | 730.00 (370.00, 1180.00) | **<0.001** |
| Annual household income (%) |  |  | **<0.001** |
| Under $44,999 | 21235 (51.5) | 4525 (72.6) |  |
| $45,000 to $74,999 | 11892 (28.8) | 1387 (22.3) |  |
| $75,000 and over | 8120 (19.7) | 319 (5.1) |  |
| Marital status (%) |  |  | **<0.001** |
| Married/cohabiting | 24364 (59.1) | 3125 (50.2) |  |
| Widowed/divorced/separated | 7344 (17.8) | 2612 (41.9) |  |
| Never married | 9539 (23.1) | 494 (7.9) |  |
| Education level (%) |  |  | **<0.001** |
| Under high school | 10420 (25.3) | 2368 (38.0) |  |
| High school or equivalent | 9768 (23.7) | 1575 (25.3) |  |
| Above high school | 21059 (51.1) | 2288 (36.7) |  |
| BMI | 27.78 (24.06, 32.31) | 27.45 (23.96, 31.58) | **<0.001** |
| Diabetes (%) |  |  | **<0.001** |
| No | 36434 (88.3) | 4556 (73.1) |  |
| Borderline | 776 (1.9) | 176 (2.8) |  |
| Yes | 4037 (9.8) | 1499 (24.1) |  |
| Hypertension = Yes (%) | 12073 (29.3) | 3699 (59.4) | **<0.001** |
| Weak/failing kidneys = Yes (%) | 979 (2.4) | 465 (7.5) | **<0.001** |
| Total Cholesterol (mmol/L) | 4.91 (4.24, 5.64) | 4.94 (4.19, 5.72) | 0.088 |
| Abbreviations: 25(OH)D = 25-hydroxyvitamin D; BMI = Body mass index. | | | |
