# Supplementary material for: Age-specific benefits of Vitamin D and its association with mortality
Source: PLoS One. 2025 Aug 29;20(8):e0330959. doi: 10.1371/journal.pone.0330959 (PMC12396682; doi:10.1371/journal.pone.0330959)
Supplement: S5 Table — (DOCX) [file pone.0330959.s013.docx]

| **Characteristic** | **Unweighted** | | **Weighted** | |
| --- | --- | --- | --- | --- |
|  | **HR (95% CI)** | **p-value** | **HR (95% CI)** | **p-value** |
| Sex |  |  |  |  |
| Male |  |  |  |  |
| Deficiency (25(OH)D <50 nmol/L) | reference |  | reference |  |
| Insufficiency (50 nmol/L≤25(OH)D ≤75 nmol/L) | 0.73 (0.67, 0.79) | **<0.001** | 0.73 (0.65, 0.81) | **<0.001** |
| Sufficiency (25(OH)D >75 nmol/L) | 0.67 (0.61, 0.74) | **<0.001** | 0.67 (0.58, 0.76) | **<0.001** |
| Female |  |  |  |  |
| Deficiency (25(OH)D <50 nmol/L) | reference |  | reference |  |
| Insufficiency (50 nmol/L≤25(OH)D ≤75 nmol/L) | 0.74 (0.67, 0.81) | **<0.001** | 0.70 (0.63, 0.77) | **<0.001** |
| Sufficiency (25(OH)D >75 nmol/L) | 0.67 (0.61, 0.74) | **<0.001** | 0.61 (0.55, 0.69) | **<0.001** |
| 18-39 |  |  |  |  |
| Deficiency (25(OH)D <50 nmol/L) | reference |  | reference |  |
| Insufficiency (50 nmol/L≤25(OH)D ≤75 nmol/L) | 1.05 (0.79, 1.39) | 0.761 | 0.98 (0.69, 1.39) | 0.896 |
| Sufficiency (25(OH)D >75 nmol/L) | 0.84 (0.58, 1.24) | 0.386 | 0.72 (0.44, 1.17) | 0.184 |
| 40-59 |  |  |  |  |
| Deficiency (25(OH)D <50 nmol/L) | reference |  | reference |  |
| Insufficiency (50 nmol/L≤25(OH)D ≤75 nmol/L) | 0.65 (0.56, 0.76) | **<0.001** | 0.61 (0.50, 0.73) | **<0.001** |
| Sufficiency (25(OH)D >75 nmol/L) | 0.59 (0.49, 0.72) | **<0.001** | 0.55 (0.44, 0.70) | **<0.001** |
| 60 and over |  |  |  |  |
| Deficiency (25(OH)D <50 nmol/L) | reference |  | reference |  |
| Insufficiency (50 nmol/L≤25(OH)D ≤75 nmol/L) | 0.73 (0.69, 0.79) | **<0.001** | 0.73 (0.67, 0.80) | **<0.001** |
| Sufficiency (25(OH)D >75 nmol/L) | 0.69 (0.64, 0.74) | **<0.001** | 0.67 (0.61, 0.74) | **<0.001** |
| Abbreviations: 25(OH)D = 25-hydroxyvitamin D; BMI = Body mass index. HRs were adjusted for age, sex, race, annual household income, marital status, education level, BMI, diabetes, hypertension, weak/failing kidneys, and total cholesterol. | | | | |
