# Supplementary material for: Age-specific benefits of Vitamin D and its association with mortality
Source: PLoS One. 2025 Aug 29;20(8):e0330959. doi: 10.1371/journal.pone.0330959 (PMC12396682; doi:10.1371/journal.pone.0330959)
Supplement: S6 Table — (DOCX) [file pone.0330959.s014.docx]

|  | **Unweighted** | | **Weighted** | |
| --- | --- | --- | --- | --- |
| **Underlying Leading Cause of Death** | **HR (95% CI)** | **p-value** | **HR (95% CI)** | **p-value** |
| Diseases of heart |  | | | |
| Deficiency (25(OH)D <50 nmol/L) | reference | | reference | |
| Insufficiency (50 nmol/L≤25(OH)D ≤75 nmol/L) | 1.28 (0.66, 2.48) | 0.471 | 1.25 (0.62, 2.52) | 0.542 |
| Sufficiency (25(OH)D >75 nmol/L) | 0.87 (0.32, 1.15) | 0.782 | 0.65 (0.14, 2.94) | 0.575 |
| Malignant neoplasms |  | | | |
| Deficiency (25(OH)D <50 nmol/L) | reference | | reference | |
| Insufficiency (50 nmol/L≤25(OH)D ≤75 nmol/L) | 0.43 (0.19, 0.98) | **0.044** | 0.35 (0.11, 1.11) | 0.075 |
| Sufficiency (25(OH)D >75 nmol/L) | 0.50 (0.18, 1.39) | 0.181 | 0.17 (0.04, 0.74) | **0.018** |
| Chronic lower respiratory diseases | number of events = 0 | | | |
| Accidents |  | | | |
| Deficiency (25(OH)D <50 nmol/L) | reference | | reference | |
| Insufficiency (50 nmol/L≤25(OH)D ≤75 nmol/L) | 1.08 (0.54, 2.15) | 0.835 | 0.98 (0.39, 2.49) | 0.968 |
| Sufficiency (25(OH)D >75 nmol/L) | 1.39 (0.65, 2.95) | 0.395 | 1.35 (0.52, 3.49) | 0.539 |
| Cerebrovascular diseases |  | | | |
| Deficiency (25(OH)D <50 nmol/L) | reference | | reference | |
| Insufficiency (50 nmol/L≤25(OH)D ≤75 nmol/L) | 0.77 (0.16, 3.72) | 0.743 | 0.38 (0.07, 1.99) | 0.251 |
| Sufficiency (25(OH)D >75 nmol/L) | 0.35 (0.03, 3.92) | 0.394 | 1.03 (0.12, 8.53) | 0.98 |
| Alzheimer’s disease | number of events = 0 | | | |
| Diabetes mellitus |  | | | |
| Deficiency (25(OH)D <50 nmol/L) | reference | | reference | |
| Insufficiency (50 nmol/L≤25(OH)D ≤75 nmol/L) | 0.55 (0.14, 2.23) | 0.402 | 0.69 (0.16, 2.97) | 0.619 |
| Sufficiency (25(OH)D >75 nmol/L) | 1.17 (0.24, 5.70) | 0.844 | 0.58 (0.12, 2.92) | 0.51 |
| Influenza and pneumonia |  | | | |
| Deficiency (25(OH)D <50 nmol/L) | reference | | reference | |
| Insufficiency (50 nmol/L≤25(OH)D ≤75 nmol/L) | 3.28 (0.00, ∞) | 0.997 | 2.91 (0.29, 29.5) | 0.366 |
| Sufficiency (25(OH)D >75 nmol/L) | 1.97 (0.00, ∞) | 1.0 | 0.97 (0.18, 5.14) | 0.976 |
| Nephritis, nephrotic syndrome and nephrosis |  | | | |
| Deficiency (25(OH)D <50 nmol/L) | reference | | reference | |
| Insufficiency (50 nmol/L≤25(OH)D ≤75 nmol/L) | 0.00 (0.00, ∞) | 1.0 | 0.00 (0.00, 0.00) | <0.001**^#^** |
| Sufficiency (25(OH)D >75 nmol/L) | 0.00 (0.00, ∞) | 1.0 | 0.00 (0.00, 0.00) | <0.001**^#^** |
| All other causes |  | | | |
| Deficiency (25(OH)D <50 nmol/L) | reference | | reference | |
| Insufficiency (50 nmol/L≤25(OH)D ≤75 nmol/L) | 1.31 (0.84, 2.03) | 0.234 | 1.30 (0.80, 2.12) | 0.288 |
| Sufficiency (25(OH)D >75 nmol/L) | 0.72 (0.37, 1.39) | 0.326 | 0.64 (0.30, 1.33) | 0.231 |
| Abbreviations: HR = Hazard ratios; CI = Confidence interval; 25(OH)D = 25-hydroxyvitamin D. HRs were adjusted for age, sex, race, annual household income, marital status, education level, BMI, diabetes, hypertension, weak/failing kidneys, and total cholesterol. ^#^This result is unreliable, see https://stat.ethz.ch/pipermail/r-help/2008-September/174201.html | | | | |
